# Lightweight and Efficient Image Dehazing Network Guided by Transmission Estimation from Real-World Hazy Scenes

**DOI:** 10.3390/s21030960

**Published:** 2021-02-01

**Authors:** Zhan Li, Jianhang Zhang, Ruibin Zhong, Bir Bhanu, Yuling Chen, Qingfeng Zhang, Haoqing Tang

**Affiliations:** 1 Department of Computer Science, Jinan University, Guangzhou 510632, China; geekco.ikst@gmail.com (J.Z.); zrb664996211@stu2017.jnu.edu.cn (R.Z.); tqfz@jnu.edu.cn (Q.Z.); hqtang@stu2017.jnu.edu.cn (H.T.); 2 Department of Electrical and Computer Engineering, University of California, Riverside, CA 92521, USA; bhanu@ee.ucr.edu; 3 China Southern Airlines Co. Ltd., Guangzhou 510080, China; cml_699@163.com

**Keywords:** single image dehazing, transmission-guided, lightweight neural network, image restoration

## Abstract

In this paper, a transmission-guided lightweight neural network called TGL-Net is proposed for efficient image dehazing. Unlike most current dehazing methods that produce simulated transmission maps from depth data and haze-free images, in the proposed work, guided transmission maps are computed automatically using a filter-refined dark-channel-prior (F-DCP) method from real-world hazy images as a regularizer, which facilitates network training not only on synthetic data, but also on natural images. A double-error loss function that combines the errors of a transmission map with the errors of a dehazed image is used to guide network training. The method provides a feasible solution for introducing priors obtained from traditional non-learning-based image processing techniques as a guide for training deep neural networks. Extensive experimental results demonstrate that, in terms of several reference and non-reference evaluation criteria for real-world images, the proposed method can achieve state-of-the-art performance with a much smaller network size and with significant improvements in efficiency resulting from the training guidance.

## 1. Introduction

Haze refers to atmospheric smoke, dust, or moisture and is a common phenomenon in daily life. The presence of haze significantly degrades the quality of images, which can lead to poor performance for various image processing tasks, including image recognition and classification, remote sensing, and video analysis. Therefore, image dehazing has attracted significant attention as a key technology for recovering degraded images captured in bad weather.

Narasimhan et al. [1] formulated the deterioration of image quality in the following atmospheric scattering equation:

(1) I(x)=J(x)T(x)+A(1−T(x)),

where *x* is the pixel location; *I* is the observed hazy image; *J* is the true scene radiance; *T* is the transmission map, and *A* is the global atmospheric light (indicating the intensity of a light source at an infinite distance).

Image dehazing is an ill-posed inverse problem that consists of computing a desired haze-free image *J* from an observed hazy image *I*, as well as estimating atmospheric light A and transmission map *T* using (1). Traditional solutions commonly add various constraints to their optimization processes by including prior information, such as colour attenuation priors (CAPs) [2], non-local priors [3], dark channel priors (DCPs) [4], and scene-depth priors [5]. However, it is typically very time consuming to estimate prior constraints for each input image. Furthermore, the differences between introduced priors and real degradation processes often have negative effects on the final outputs, including insufficient or excessive dehazing, colour distortions, halos, and artifacts. To alleviate these issues, recent learning-based methods have attempted to train deep neural networks (DNNs) from a set of examples [6,7,8,9,10,11,12,13,14] without formulating prior knowledge. Such methods include DehazeNet [6], all-in-one dehazing (AOD) [8], Cycle-Dehaze (Cycle) [9], proximal dehazing network (PDN) [10], and grid dehazing network (GDN) [11]. A well-trained DNN can perform dehazing and enhancing operations on real images with higher efficiency and superior visual effects than prior-based methods. However, it is difficult to derive optimal network parameters without any guidance from prior knowledge because the features extracted by a network may not always be related to the degradation caused by haze [12].

As indicated in (1), transmission map *T* is a key factor of the solution. It is beneficial and practical to introduce transmission maps as prior information to guide the training of a dehazing network. However, it is difficult to obtain real transmission maps as ground-truth information for natural images, and such maps are rare in datasets available on the internet. Therefore, as an alternative, many previous works have used either depth-of-field (DOF) maps or semantic segmentation maps [7,8,10,11,12,13]. Otherwise, a constant transmission rate can be applied to an entire image for simulated experiments [6]. However, such practices are inconsistent with the spatially variable transmission found in real-world hazy scenes.

**Figure 1:**
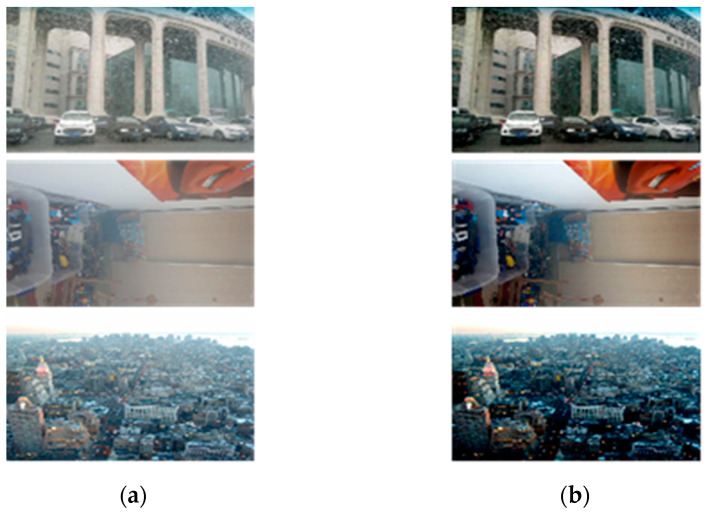
Sample image dehazing results using the proposed transmission-guided lightweight network (TGL-Net). (**a**) Hazy input; (**b**) dehazed output.

In this work, a transmission-guided lightweight network (TGL-Net) for fast natural image dehazing is proposed. We take advantage of the effectiveness of DNNs and introduce the transmission map as a prior information to guide the efficient training of the network. Notably, this paper presents a feasible solution for introducing priors obtained from non-learning- based image processing techniques as guidance for training DNNs. Instead of producing the transmission from predefined DOF maps, we apply a filter-based DCP (F-DCP) method [15] to estimate transmission maps from input hazy images automatically, thereby avoiding additional manual calibration or the collection of transmission information. By introducing transmission guidance, the proposed TGL-Net can achieve state-of-the-art (SOTA) performance with fewer parameters and smaller network dimensions, as well as faster training and processing speed. Figure 1 presents some sample results for real-world hazy images processed by TGL-Net. Additional experimental results are presented in Section 4.

The main contributions of this paper are:

- We propose a lightweight network, TGL-Net, based on a condensed residual encoder–decoder structure with skip connections for the fast dehazing of real-world images.
- To compute transmission errors without additional information for real-world hazy images, a reference transmission map is automatically estimated from a hazy image using a non-learning-based method F-DCP, which is a transmission-improved DCP method based on filters.
- A double-error loss function is introduced to combine the errors of a transmission map and dehazed output to supervise the training process of the network. With guidance from the proposed loss function, prior information is introduced into the model, yielding outputs with richer details and information.
- Both natural images and synthetic datasets are used for training the TGL-Net to make the model more applicable to real-world image dehazing and to achieve more rapid convergence during the training process.

The remainder of this paper is organized as follows. In Section 2, we review several related works. In Section 3, the TGL-Net model and proposed method are discussed in detail. In Section 4, qualitative and quantitative comparisons, as well as experimental results, are presented. In Section 5, our conclusions are summarized.

## 2. Related Work

In this section, we briefly review previous methods for single-image dehazing that range from traditional image enhancement techniques and recovery-based algorithms to the most recent learning-based methods.

Traditional enhancement methods attempt to address the dehazing problem using various image enhancement techniques, such as histogram processing [16] and saturation-based processing [17], to enhance the visual quality of output images. Most recovery methods follow the physical atmospheric scattering model and attempt to introduce various reasonable prior assumptions as constraints to regularize the ill-posed inverse problem. Tan [18] attempted to recover per-patch contrast based on the observation that haze significantly decreases contrast in colour images. Inspired by statistical results suggesting that the minimum intensities of RGB colour channels in a natural haze-free image are close to zero, He et al. [4] proposed a DCP-based method to estimate transmission maps. Meng et al. [19] introduced a specific boundary constraint to estimate more accurate transmission maps. Fattal et al. [20] estimated the albedo of a scene by relying on the assumption that transmission and surface shading are locally uncorrelated. Considering the differences between the brightness and saturation of pixels in hazy images, Zhu et al. [2] proposed a linear CAP. Because the pixels in a given cluster are often non-local, Berman et al. [3] introduced a non-local prior to recover both distance maps and haze-free images. Recently, Chen et al. [15] improved the original DCP dehazing method by designing several filters (F-DCP) to refine transmission maps. They then applied a piecewise constrained function to preserve colour fidelity.

In recent years, convolutional neural networks (CNNs) and deep learning techniques have been widely used for image dehazing. Cai et al. [6] constructed an end-to-end CNN-based haze-removal system called DehazeNet to learn mappings between input hazy images and corresponding transmission maps. Ren et al. [7] developed a multi-scale CNN with coarse-scale and fine-scale networks to predict holistic transmission maps. Instead of computing transmission maps and atmospheric light values separately, Li et al. [8] designed a lightweight all-in-one dehazing (AOD) network incorporating an atmospheric scattering model. More recently, Deniz et al. [9] enhanced the Cycle-GAN model by combining cycle consistency with perceptual loss and proposed the cycle model, which requires neither paired samples of hazy and haze-free images nor any parameters of atmospheric scattering model for the training. A proximal dehazing network (PDN) was constructed by Yang et al. [10] to integrate haze imaging model constraints and image prior learning into a single network. Liu et al. [11] proposed an end-to-end trainable grid dehazing network (GDN) for single-image dehazing. This method does not rely on an atmospheric scattering model. Chen et al. [12] designed a CNN called the patch map selection network (PMS-Net) to select patch sizes corresponding to each pixel adaptively and automatically. Li et al. [14] proposed a semi-supervised image dehazing (SSID) network, which incorporates both a supervised learning branch and an unsupervised learning branch, therefore it can be trained on both the synthetic data and real-world images.

Although the concept of incorporating transmission loss into cost functions has been applied in several image dehazing methods [7,8,10,12,13], our work differs significantly in that we estimate guiding transmission maps using a non-learning-based method for real hazy images instead of generating simulated transmission maps from depth data and haze-free images. This allows our network to be trained using not only synthetic data, but also real hazy images without depth data or transmission priors. Table 1 summarizes the properties of different dehazing networks. Most other image dehazing models are not trained on real datasets but on synthetic datasets if they are guided by transmissions, such as [6,10,12,13]. The transmission maps used by these models are computed from depth data, because existing open datasets, such as the NYU [21], Middlebury Stereo [22], and RESIDE [23] datasets, only provide depth data for clear haze-free images and not for real-world hazy images. However, because real hazy datasets, such as the NTIRE challenge datasets [24,25,26], provide real-world hazy and haze-free image pairs without any information regarding transmission, previous models trained using real datasets can rarely be guided by transmission priors. In Table 1, two other methods, Cycle [9] and SSID [14], which can be trained on real-world hazy images, are unsupervised or semi-supervised methods. Both of them have much larger model size, more parameters, and longer average runtime compared with ours, as we detailed in Section 4. To the best of our knowledge, ours is the first lightweight and efficient transmission-guided image dehazing network that can be trained using real hazy images.

## 3. Proposed Method

In the following subsections, we present the details of the proposed network architecture, loss functions, and training methodology.

### 3.1. Architecture

We design the proposed TGL-Net architecture based on a very deep residual encoder–decoder network (RED-Net) [27] with a symmetric structure and skip connections, which was originally applied to image restoration tasks, such as denoising and super-resolution. However, our network is very lightweight and has far fewer layers and parameters than RED-Net.

**Figure 2:**
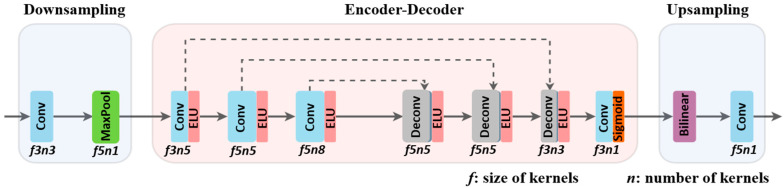
Symmetric architecture of TGL-Net consists of three parts: downsampling, encoder–decoder and upsampling. It is based on a condensed residual encoder–decoder structure with skip connections (dotted lines).

TGL-Net is divided into three phases: downsampling, encoder–decoder, and upsampling. This structure is illustrated in Figure 2. The parameter settings related to the network structure are listed in Table 2, including the sizes and channels of inputs for every layer (presented as *h* × *w* × *c*), as well as the sizes and numbers of kernels (presented as *f* × *f* × *n*). A stride of one is used for all convolutional and deconvolutional operations.

#### 3.1.1. Downsampling

The downsampling phase is composed of a convolutional layer and a max-pooling layer. Image features are extracted from the convolutional layer, and a maximum pooling operation with a stride of five is used. Following this pooling operation, feature maps with heights and widths equal to one-fifth of the input image dimensions are produced. Therefore, the total amount of input data is reduced by 25 times to improve the computational efficiency of the network. Related experiments on the effects of downsampling and upsampling layers are presented in our ablation study discussed in Section 4.3.2.

#### 3.1.2. Encoder–Decoder

We apply a condensed encoder–decoder connection with three pairs of convolutional and deconvolutional layers as the main phase of the proposed TGL-Net for feature extraction and transmission estimation. In the encoder, convolutional layers are used to extract image features and eliminate noise simultaneously. In the decoder, deconvolutional layers are used to recover the details of transmission maps. The sizes of convolution kernels in the encoder are 3 × 3, 5 × 5, and 5 × 5 in sequence, and the corresponding deconvolution kernels are the same, but in reverse order. Zero padding is applied to ensure that the sizes of the output feature maps from each layer are the same.

We retain the network structure of symmetrically linked convolutional and deconvolutional layers with skip connections from the original RED-Net [27] because this structure takes advantage of residual network characteristics [28], including rapid convergence and high accuracy. Specifically, using skip connections, signals can be propagated directly to subsequent layers. Therefore, the risk of the common problems in DNNs called vanishing gradients or gradient explosions [29] is reduced. Furthermore, the information extracted from input images is transferred from the convolutional layers to the deconvolutional layers via skip connections, which is helpful for preserving useful image features and details.

However, instead of constructing a very deep network like RED-Net, we construct a lightweight network. To downscale TGL-Net, we reduce the encoder–decoder phase of the original RED-Net from 20 or 30 layers to six layers, thereby significantly improving computational efficiency. The experimental results presented in Section 4 reveal that TGL-Net has fewer parameters and shorter inference times than most SOTA haze removal networks with comparable dehazing effects.

**Figure 3:**
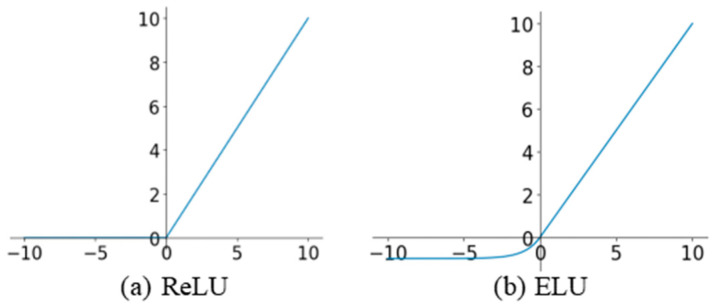
Rectified linear unit (ReLU) and exponential linear unit (ELU) activation functions. (**a**) ReLU; (**b**) ELU.

Additionally, instead of using the rectified linear unit (ReLU) activation function from RED-Net, an exponential linear unit (ELU) activation function is applied to each encoder and decoder layer. This avoids the excessive number of “dead nodes” introduced by ReLU activation, while maintaining the positive linear aspects of ReLU activation to avoid the vanishing gradient problem, as shown in Figure 3. Moreover, the mean value of ELU outputs is close to zero, which results in faster convergence for training [30]. Therefore, the ELU function is used to activate the encoder and decoder layers in TGL-Net. The results of our experiments indicate that the convergence of the network training is accelerated by replacing ReLU activation with ELU activation.

Following the encoder–decoder layers, a single convolution kernel is used to combine a three-channel feature map into a single-channel predicted transmission map. Next, the sigmoid function is applied as a nonlinear activation function. When an input is large or small (i.e., larger than 5 or smaller than −5), the sigmoid function has a significant chance of entering its saturation region, where the gradient value is almost zero. In such cases, according to the chain rule, the risk of the gradient vanishing increases with the addition of more network layers. However, the ELU units and skip connections used in our encoder–decoder layers are effective at preventing the vanishing gradient problem. The output of the nonlinear sigmoid activation function is a preliminary estimation of a reduced transmission map with a size equal to only 1/25 of that of the input image.

#### 3.1.3. Upsampling

The purpose of upsampling is to enlarge the transmission map to the same size as that of the input image. This phase is divided into two sequential steps. First, considering the computational efficiency of the entire network, bilinear interpolation is used for image expansion. On the one hand, it is well known that the nearest-neighbour interpolation suffers from mosaic effects. On the other hand, bicubic or more complicated interpolations may produce enlarged outputs with better perceptual quality, but require more computations. Moreover, in this module, the interpolation operation is followed by applying a convolutional layer to further refine the output transmission map. Therefore, we chose the effective and efficient bilinear upsampling instead of other sampling techniques.

### 3.2. Double Error Loss Function

In this paper, we propose a double-error loss function for network training that combines the errors of both transmission maps and dehazed outputs. Based on this loss function, the transmission maps estimated by traditional image restoration techniques are introduced as prior knowledge to reduce randomness and blindness during the training of TGL-Net. Furthermore, the proposed loss function enhances the details and information of dehazed outputs.

**Figure 4:**
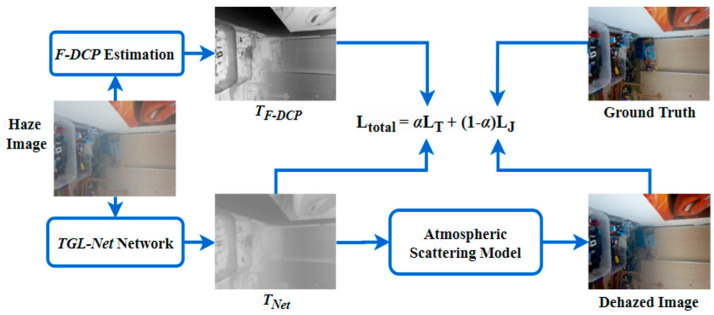
Training process for the proposed TGL-Net. *L_T_* and *L_J_* are defined in (2) and (4).

Figure 4 presents the training process of TGL-Net based on the proposed double-error loss function. To achieve reduced complexity and training time, TGL-Net estimates single-channel transmission maps, rather than haze-free images with three colour channels. Next, dehazed images are obtained by solving the equation of the atmospheric scattering model (see (1)). A transmission map estimated by the F-DCP [15] method is used as a reference for comparing the transmission map of the network output. We select the F-DCP algorithm based on its superior performance in terms of transmission estimation. In F-DCP, the initial transmission map estimated by the DCP algorithm is refined using Sobel and mean filters to enhance edge details, resulting in preferable outputs for image dehazing. Notably, a previous version of this algorithm using the Prewitt operator instead of the Sobel operator achieved the highest score among all non-learning-based methods in the NTIRE 2018 outdoor tracking challenge of [31].

The mean squared error between F-DCP transmission and its counterpart in the network output is calculated as the transmission loss LT, which is formulated as follows:

(2) $$L_{T}=\frac{1}{2WH}\sum_{w=1}^{W}\sum_{h=1}^{H}{\left\|T_{Net}(I,\Theta )-T_{F-DCP}(I)\right\|}_{}^{2},$$

where *I* is an input image with a resolution of *W × H*; Θ is the set of network parameters, and *T_Net_* and *T_F-DCP_* refer to the model functions implemented by TGL-Net and by F-DCP, respectively, both of which transform input images into grayscale transmission maps with the same size of *W × H*.

Next, a dehazed image, *J_Net_*, is generated by substituting the network output transmission map *T_Net_*(*I*, Θ) into (1).

(3) $$J_{Net}=\frac{I-A}{T_{Net}(I,\Theta )}+A,$$

where the atmospheric light *A* can be set to *A* = (1, 1, 1), assuming that the initial input image is globally white balanced [32]. In (4), *J_Net_* is compared to the reference haze-free ground-truth image *J_GT_* to obtain dehazing loss *L_J_*.

(4) $$L_{J}=\frac{1}{2CWH}\sum_{c=1}^{C}\sum_{w=1}^{W}\sum_{h=1}^{H}{\left\|J_{Net}-J_{GT}\right\|}_{}^{2},$$

where *C* refers to the number of channels in the input image *I*, and both *L_T_* and *L_J_* are standard two-norm distances.

The total loss function combines transmission loss *L_T_* and dehazing loss *L_J_* linearly, as shown in (5).

(5) $$L_{total}=\alpha L_{T}+(1-\alpha )L_{J},$$

where *α* is a tradeoff parameter. During the training process, total loss *L_total_* decreases, which indicates that the errors of both the transmission map and dehazed image decrease. However, as dehazing is the aim of our task, while transmission information is used as an auxiliary, a reasonable constraint of 0 < *α* < 0.5 is recommended. With guidance from the image restoration algorithm F-DCP, TGL-Net produces transmission maps more effectively and efficiently for the task of image dehazing. Additionally, for TGL-Net, which is trained using both synthetic and natural hazy images, the process of dehazing is less dependent on prior knowledge regarding an artificial hypothesis than traditional recovery-based methods, meaning it is more practical and robust for real-world applications.

### 3.3. Network Training

The training process for TGL-Net is summarized in Algorithm 1. The network is optimized by minimizing the total loss function *L_total_*.
**Algorithm 1** Training algorithm**Set:**  $n_{b}\leftarrow$ the batch size  Θ← the set of network parameters  A← global atmospheric light  eps← error tolerance threshold **Input:**  Sample hazy examples $I=\left\{i^{\left(1\right)},\ldots ,i^{\left(n_{b}\right)}\right\}$  Sample haze-free examples $J_{GT}=\left\{j_{GT}^{\left(1\right)},\ldots ,j_{GT}^{\left(n_{b}\right)}\right\}$ 1: **repeat** 2:  $T_{F-DCP\;}\left(I\right)\leftarrow \;$*F-DCP*(*I*), transmission map produced from F-DCP; 3:  $T_{Net\;}\left(I,\Theta \right)\leftarrow TGL\left(I,\Theta \right)$, transmission map produced from TGL-Net; 4:  Calculate the $L_{T}$ using $T_{F-DCP}$ and $T_{Net}\left(I,\Theta \right)$ by Equation (2); 5:  Calculate $J_{Net}$ by Equation (3); 6:  Calculate the $L_{J}$ using $J_{Net}$ and $J_{GT}$ by Equation (4); 7:  Bring $L_{T}$ and $L_{J}$ into Equation (5) to get $L_{total}$; 8:  Update TGL-Net by descending the gradient of $L_{total}$; 9: **until**
$L_{total}<eps$


## 4. Experimental Results

### 4.1. Experimental Setting

#### 4.1.1. Datasets

We use the NYU depth dataset [21] to generate synthetic hazy images and included the NTIRE challenge dataset [24,25,26], the Utexas LIVE defogging dataset [33], and 48 natural hazy images from the internet (Internet-48) as real-world image sets. Among these datasets, the NYU and NTIRE datasets are used for training, while the NTIRE, LIVE, and Internet-48 datasets are used for testing.

The NYU dataset contains abundant indoor haze images and corresponding depth maps. Because the transmission rate in a scene affects the fraction of light that reaches the camera sensor, it is a distance-dependent factor. Therefore, transmission *T*(*x*) at location *x* is theoretically defined as a function of DOF *d*(*x*) as follows [1]:

(6) $$T(x)=e^{-\beta d(x)},$$

where *β* represents the attenuation coefficient of the atmosphere, and *d* is the scene depth. The large value of *β* is related to a dense fog in a scene, while *β = 0* corresponds to a haze-free image. Using ground-truth haze-free images and corresponding depth metadata from the NYU dataset, we generated 16,038 synthetic hazy images with various levels of fog densities by randomly setting *β ∈* [0.5, 1.5] in (6) and atmospheric light *A* *∈* [0.7, 1.0] in (1). All synthesized images have a resolution of 480 × 640. We used 15,988 images for training and 50 images for validation.

The NTIRE 2018 dataset contains haze-free and corresponding natural hazy image pairs, with 60 images for training, 10 for validation, and 10 for testing. These images were all captured in real-world indoor and outdoor environments in the presence and absence of haze. Two professional fog/haze machines were used to generate dense vapour [24,25,26]. To enlarge the training dataset, we implemented a multi-scale cropping strategy [34] to preprocess the 60 training pairs and obtained 4860 pairs with a normalized resolution of 480 × 640. We combine these real-world image pairs with the 15,988 synthetic image pairs generated from the NYU dataset for training.

For this training process, we use a synthetic haze set generated from the NYU dataset (training set A), and an augmented real haze set generated from the NTIRE 2018 dataset (training set B). These two sets are combined to train a model called TGL-Net-Mix, allowing it to learn the real degradation characteristics of natural hazy images while being both practical and robust. In the ablation experiments described in Section 4.3, we also train a reference network called TGL-Net-Syn using only training set A.

#### 4.1.2. Implementation Details

The proposed network was trained on an Nvidia RTX-2080Ti GPU and an Intel i9-9900k CPU using the Tensorflow framework. However, a desktop with an Intel Core i5 CPU, 8 GB of memory, and Windows 10 OS was used for most of the testing. We used a standard ELU function with the default parameter in TensorFlow, i.e., “tf.nn.elu()”. We set *α* = 0.2 for the total loss function in (5) and trained the network for more than 10 epochs until it converged. For the training process, we adopted the Adam optimizer with an initial leaning rate of 0.001 and batch size of 16.

#### 4.1.3. Quality Measures

Several assessment indices are used to compare SOTA dehazing models, namely the peak signal-to-noise ratio (PSNR) and the structural similarity index (SSIM) for evaluations based on reference haze-free images, the average gradient (AG) [15], image entropy (IE) [35], the fog-aware density evaluator (FADE) [33] provided with the LIVE dataset, blind image quality measure of enhanced images (BIQME) [36], and patches-to-pictures quality predictor (PaQ2PiQ) [37] for non-reference image quality evaluation. AG is related to the edges and variance in an image and is defined by (7). A larger value of AG indicates that more details are recovered. As calculated using (8), IE represents the amount of information contained in an image based on information theory. The FADE varies with fog density conditions. A smaller FADE value corresponds to less fog. Because haze and fog decrease the visibility of scenes, dehazing techniques should increase IE and AG values and decrease FADE values.

(7) $$AG=\frac{1}{\left(W-1)\times (H-1\right)}\sum_{w=1}^{W-1}\sum_{h=1}^{H-1}\sqrt{\frac{1}{2}(\Delta I_{w}^{}{}^{2}+\Delta I_{h}^{}{}^{2})},$$

(8) $$IE=\sum_{r=0}^{L-1}\frac{H_{r}}{W\times H}log\frac{H_{r}}{W\times H},$$

where *I* indicates the image to be evaluated; (*w*, *h*) are the pixel coordinates; *W* and *H* are the width and height of the image, respectively; *L* is the maximum grayscale value; *H_r_* is the number of pixels whose grayscale value is *r*; and △*I_w_* and △*I_h_* are first-order gradients in the horizontal and vertical directions, respectively.

BIQME and PaQ2PiQ are two learning-based non-reference metrics proposed for blind image quality evaluation. BIQME [36] extracts 17 features by analyzing contrast, sharpness, brightness etc., and it predicts evaluations on image quality by training a regression module. Meanwhile, PaQ2PiQ [37] evaluates both global and local image quality by training a modified ResNet-18 [28] on a new dataset. This training dataset comprises pictures and patches associated with human perceptual quality judgements, i.e., mean opinion score (MOS). Larger values of BIQME in the range [0, 1] and PaQ2PiQ in the range [0, 100] indicate better image quality.

### 4.2. Comparisons

#### 4.2.1. Evaluations on NTIRE Datasets

The NTIRE challenge released two datasets of indoor and outdoor hazy images in 2018 and one image set with extremely dense haze in 2019 for dehazing competitions. Most NTIRE images have large sizes, ranging from approximately 2000 × 2000 to 4000 × 4000. The NTIRE 2018 dataset is used for testing after being separated into NTIRE18Val-10 (10 images in NTIRE 2018 validation set), NTIRE18Train-60 (60 images in NTIRE 2018 training set), and NTIRE18-20 (20 images from the NTIRE 2018 validation and testing sets) datasets to ensure that no training images were included in the testing set. Furthermore, to evaluate the performances of dehazing models for scenes with extremely dense haze, all 10 images from the validation and testing sets of the NTIRE 2019 dataset [26] (hereinafter referred to as NTIRE19-10) are tested.

We compare the proposed TGL-Net to three SOTA dehazing networks, namely DehazeNet [6], AOD [8], and Cycle [9]. Additionally, because TGL-Net is trained with guidance from transmission maps estimated by F-DCP [15], F-DCP is also considered as a baseline, even though it is not a learning-based approach.

**Figure 5:**
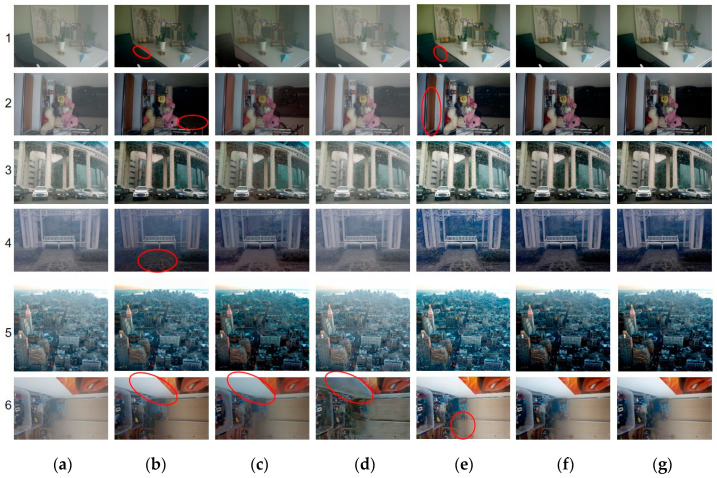
Qualitative comparisons of real-world hazy images. (**a**) Hazy Inputs; (**b**) DehazeNet [6]; (**c**) all-in-one dehazing (AOD) [8]; (**d**) Cycle [9]; (**e**) filter-refined dark-channel-prior (F-DCP) [15]; (**f**) TGL-Net-Syn; (**g**) TGL-Net-Mix.

Figure 5 presents some examples for qualitative comparisons. Some noteworthy local areas are highlighted with red circles. In general, Cycle produces outputs with insufficient dehazing and many negative artifacts, particularly in images with a small depth of field, such as indoor image No. 6 in the last row in Figure 5d. DehazeNet tends to darken input images, which is very apparent in images nos. 1, 2, and 4 in Figure 5b (see the red-circled regions in these images). AOD outperforms DehazeNet, but still slightly darkens some white areas, such as the wall in image no. 6 in Figure 5c. F-DCP recovers images with brighter colours, but there are vertical stripes in some of the outputs of F-DCP (see the red-circled regions in images nos. 1, 2, and 6 in Figure 5e). Because F-DCP assumes that the DOF is larger at the top of a natural scene image and smaller at the bottom, images that do not satisfy this constraint will result in such artifacts. In general, visual comparisons reveal that the two TGL-Nets have dehazing performance comparable to that of DehazeNet and AOD, but with better colour fidelity (see the preservation of the white wall in image no. 6). Furthermore, although the proposed TGL-Nets are trained with guidance from F-DCP transmission maps, they do not learn the vertical stripe artifacts present in the F-DCP outputs. It is difficult to distinguish Figure 5f,g visually. However, the training of TGL-Net-Syn converged more slowly and unsteadily than that of TGL-Net-Mix, which will be discussed in a later section.

Table 3 lists the PSNR and SSIM values for the NTIRE 2018 dataset. Numbers in red, blue, and green indicate the first-, second-, and third-best results, respectively. The Cycle network produces images with an original resolution of 256 × 256 and then upsamples these images using a Laplacian post-processing technique [9], but most images in the NTIRE 2018 dataset are much larger in size with a resolution of more than 2000 × 2000. Laplacian-enlarging changes pixel intensities, which leads to deviations in PSNR and SSIM values, so for a fair comparison we do not compute these two indices for the Cycle network. Moreover, the NTIRE 2018 training set was used for training TGL-Net-Mix, so it could not be used to test this model. Table 3 shows that our models exhibit competitive performance on the NTIRE 2018 datasets, with slight advantages on the NTIRE18Val-10 dataset.

Additional experiments were conducted based on non-reference evaluations because most real-world hazy images do not have corresponding haze-free images acting as a ground-truth for evaluation. Table 4 lists the values of AG, IE, FADE, BIQME, and PaQ2PiQ for the network models for two NTIRE datasets. These metrics are used for comprehensive blind image quality evaluations on contrast, visibility, sharpness, details, and so on, as detailed in Section 4.1.3. Among these models, TGL-Net-Mix achieves the highest AG values for both sets, with F-DCP coming in second place. Because F-DCP enhances the edges and details of transmission maps using a set of filters, and TGL-Net-Mix is trained with guidance from F-DCP transmissions, these two methods significantly improve edge reconstruction, as indicated by the AG values. Additionally, our network has the smallest FADE value, second-best BIQME value, and third-best IE and PaQ2PiQ values in the NTIRE18-20 dataset related to moderate-density fog. It also has the largest PaQ2PiQ value, second-best IE, FADE, and BIQME values in the NTIRE19-10 dense-haze image set. These results suggest that TGL-Net-Mix exhibits preferable dehazing performance for common natural haze scenes, but it is slightly inferior to AOD for scenes with extremely dense fog.

#### 4.2.2. Evaluations on Other Datasets

For additional evaluations on various real hazy scenes, 500 images from the Utexas LIVE image defogging dataset [33], which are referred to as LIVE-500, and the Internet-48 dataset are also considered. Both sets contain natural foggy images of outdoor scenes with smaller sizes than the NTIRE images. Most images in the Internet-48 dataset have been frequently used in other dehazing studies. Several recent DNNs, namely DehazeNet [6], AOD [8], PDN [10], and GDN [11], as well as two prior-based dehazing methods, namely CAP [2] and F-DCP [15], are included for comparison.

**Figure 6:**
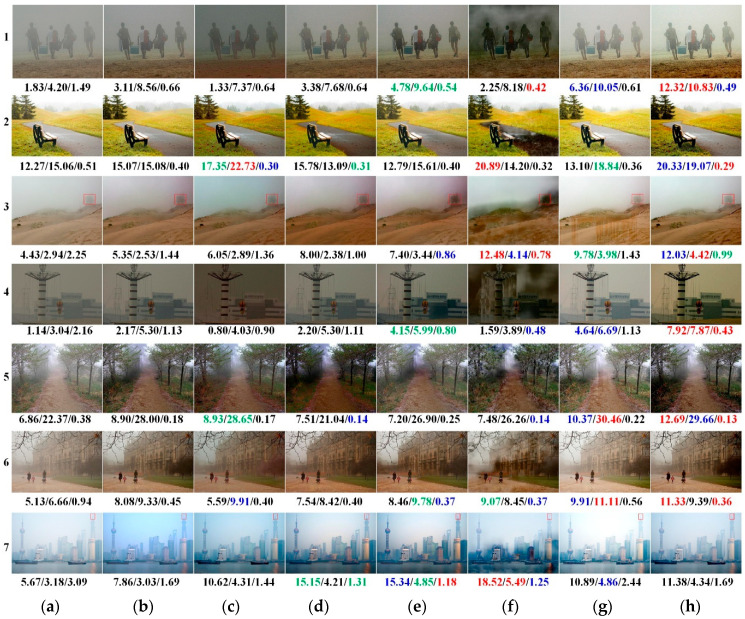
Qualitative comparisons on the LIVE foggy outdoor dataset [33] with average gradient (AG), image entropy (IE), and the fog-aware density evaluator (FADE) values listed below each image. The 1st, 2nd, and 3rd winners of each measurement are displayed in red, blue, and green colors, respectively. (**a**) Hazy Inputs; (**b**) DehazeNet [6]; (**c**) AOD [8]; (**d**) colour attenuation priors (CAP) [2]; (**e**) proximal dehazing network (PDN) [10]; (**f**) grid dehazing network (GDN) [11]; (**g**) F-DCP [15]; (**h**) TGL-Net-Mix.

Figure 6 presents some examples for qualitative comparison. For reference, the AG, IE, and FADE values are listed in sequence below each image. Images nos. 1 and 4 in Figure 6c, as well as image no. 5 in Figure 6d, are darkened due to excessive dehazing of nearby objects by AOD and CAP. Similar to Figure 5, there are vertical stripes on the sandy ground in image no. 3 and on the distant trail in image no. 5 in Figure 6g based on unrealistic assumptions regarding the DOF by F-DCP. Additionally, obvious halos can be observed in regions where the DOF jumps in the output images generated by GDN, such as the sky in images no. 1, 3, 4, and 7, as well as the close pathway in image no. 2 in Figure 6f. Additionally, the colour of the sky in image no. 3 is changed from greyish to light pink or light blue in Figure 6b–f, indicating the introduction of colour distortions.

In general, the proposed TGL-Net-Mix produces outputs with preferable values of AG, IE, and FADE for images in Figure 6, except for image no. 7 in the last line, due to its good performance on enhancing details and removing haze for most real-world foggy images. With respect to the three criteria below each image, GDN is the second-best one with several red numbers. However, it has the worst visual perception by human subjects due to the greater number of halos in sky regions and artifacts at the edges. As AG, IE, and FADE values are measurements corresponding to edges, details, and visibility, respectively, they are sensitive to image contrasts and artifacts. Therefore, halos and artifacts sometimes lead to an illusion of improvement in performances due to an increasement of these measurements. Another similar case is F-DCP, which introduces vertical stripes in the outputs of image nos. 3 and 5 in Figure 6g, but achieves better results as regards IE and AG values.

**Figure 7:**
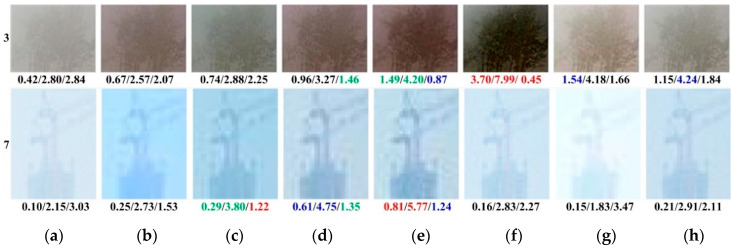
Local areas from red boxes in images nos. 3 and 7 in Figure 6 with local AG, IE, and FADE values listed below each image. The 1st, 2nd, and 3rd winners of each measurement are displayed in red, blue, and green colors, respectively. (**a**) Hazy Inputs. (**b**) DehazeNet [6]. (**c**) AOD [8]. (**d**) CAP [2]. (**e**) PDN [10]. (**f**) GDN [11]. (**g**) F-DCP [15]. (**h**) TGL-Net-Mix.

To highlight additional details, Figure 7 presents enlarged views of the distant plants in image no. 3 and the top of a remote tower in image no. 7 from the red boxes in Figure 6. Local average AG, IE, and FADE values are shown below each image. Image no. 3 in the first rows of Figure 7b,d–g, as well as image no. 7 in the second row of Figure 7b, exhibit colour distortions introduced by DehazeNet, CAP, PDN, GDN, and F-DCP. Additionally, artifacts exist at edges in the outputs of AOD, CAP, and PDN in the second row of Figure 7c–e. Figure 7 reveals that the dehazed outputs of the proposed TGL-Net-Mix generally have clearer edges and better visibility with fewer artifacts and less colour distortion than the outputs of the other methods, although its local quantitative indices show no significant advantages. Similar to Figure 6, better values of the three metrics below the local image no. 3 in Figure 7f and the local image no. 7 in Figure 7c,e may be caused by the color distortions and artifacts around the edges in these outputs.

Table 5 lists average metrics for different methods on the LIVE-500 and Internet-48 testing sets. However, instead of evaluating image dehazing techniques in particular, BIQME and PiQ2PaQ are proposed as general blind measures of image quality training on images with other types of degradations. Most dehazing methods have unsatisfactory values for these two criteria. Even the hazy inputs produce better PiQ2PaQ values than do dehazed outputs, such as the results of most methods on LIVE-500 except for F-DCP. In general, the qualitative comparisons and quantitative criteria values in Figure 6 and Figure 7, and Table 5 indicate that TGL-Net-Mix generates preferable results for most real-world hazy images in the LIVE dataset. However, some outputs with clear artifacts or color shifts may occasionally exhibit better metric values. As mentioned previously, AG, IE, and FADE values are sensitive to image contrast and artifacts, and they are unconnected with color fidelity. Therefore, it is possible to achieve good values for these metrics while generating colour distortions, excessive dehazing, or overly enhanced image contrast. Additionally, artifacts such as stripes and halos can increase the values of AG and IE while reducing the value of FADE in some cases. For example, the outputs of GDN have the worst halo effects, but achieve some of the best values for AG, IE, and FADE, as seen in images nos. 1, 2, 3, and 7 in Figure 6f, the first row in Figure 7f, and the small FADE values in Table 5. The results are similar for F-DCP, where the artificial vertical stripes increase its AG and IE values in Figure 6 and Table 5. TGL-Net-Mix clearly benefits from transmission guidance as more details and information are recovered, but with no vertical stripes, which is reflected in its high AG values compared to F-DCP.

**Figure 8:**
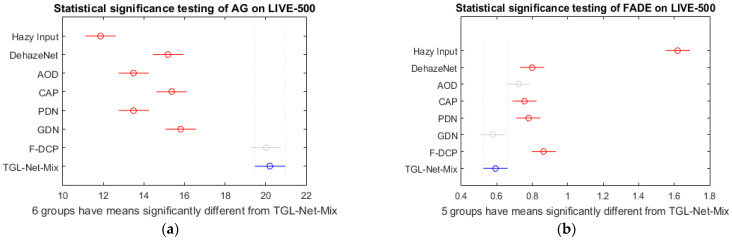
Statistical significance testing for AG and FADE measures on the dataset LIVE-500. Group of the proposed TGL-Nex-Mix is described in blue. Groups that have means significantly different from ours are described in red. The others are in grey. The centre of each circle corresponds to the mean value of 500 images of each group. (**a**) AG; (**b**) FADE.

In addition, in Table 5, TGL-Net-Mix achieves the best score of AG and the second-best score of FADE on the dataset LIVE-500. To verify whether these improvements are significant, we computed statistical significance for AG and FADE metrics on LIVE-500 using a standard function “multcompare()” in MATLAB R2015a. This testing is only carried out on the LIVE-500 dataset, because it contains sufficient number of images to make statistical conclusions, in contrast with other testing sets that we have used. Figure 8 displays the results. Groups that have means significantly different from ours are shown in red, which contain six for AG values and five for FADE values, respectively. Further, groups without significant differences from ours in Figure 8a,b are not repetitive (i.e., F-DCP w.r.t. AG, and AOD and GDN w.r.t. FADE). Therefore, the proposed network is significantly better in both of these two measures, which indicates improved performance of enhancing edges and removing fog.

#### 4.2.3. Network Size and Efficiency

We compare the number of network parameters of TGL-Net to those of several SOTA dehazing networks, namely DehazeNet [6], AOD [8], Bilinear-Net [38], PMS-Net [12] and Join-GAN [13]. Because our network uses a condensed encoder–decoder architecture based on RED-Net, the RED-20 and RED-30 networks used in [21] are also considered for comparison. The number of parameters of these networks are listed in Table 6. All models except AOD have much larger parameter sets than TGL-Net. We did not compute the numbers of parameters for PDN [10] and GDN [11]. However, based on the network architectures presented in the corresponding studies, they are supposed to have much larger sizes than TGL-Net, which can be verified based on the comparisons of average run times in Table 7.

Table 7 lists the average run times of several dehazing methods on the LIVE-500 and Internet-48 testing sets. These methods are evaluated on a desktop computer with an Intel Core i5 CPU, 8 GB of memory, and Windows 10 OS. As shown in Table 7, TGL-Net is the fastest among the seven compared dehazing methods on both test sets, meaning it significantly improves the efficiency of haze removal. Although TGL-Net has approximately twice as many parameters as AOD, it is nearly six times faster on average on the LIVE-500 and Internet-48 datasets.

In Table 8, to evaluate model efficiency for large images, we compare the average run times for 60 indoor and outdoor real hazy images with an average resolution of 2945 × 4104 from the NTIRE 2018 dataset on an Nvidia RTX-2080Ti GPU. Only DehazeNet, AOD, and TGL-Net are compared as three representative lightweight networks. As a high-efficiency model, TGL-Net is approximately 25 times faster than AOD and 1000 times faster than DehazeNet on average.

As shown in Table 6, Table 7 and Table 8, TGL-Net is a lightweight network with remarkable superiority in terms of computational efficiency.

### 4.3. Ablation Study

#### 4.3.1. Transmission Loss and Training Set

To study the contributions of transmission loss *L_T_* and real haze training sets, we trained three models, L-Net-Mix, TGL-Net-Syn, and TGL-Net-Mix. The settings for these models are listed in Table 9, in which training set A refers to a synthetic haze set generated from the NYU dataset, and training set B is an augmented real haze set generated from the NTIRE 2018 dataset (see Section 4.1). Table 9 reveals that both transmission loss and a mixed training set can improve dehazed outputs in terms of average PSNR.

Table 10 lists representative AG values for L-Net-Mix and TGL-Net-Mix. Because F-DCP refines edges in transmission maps using filters, with guidance from F-DCP transmissions, TGL-Net-Mix significantly improves the edge-related AG metric compared to L-Net-Mix, which does not use transmission guidance.

**Figure 9:**
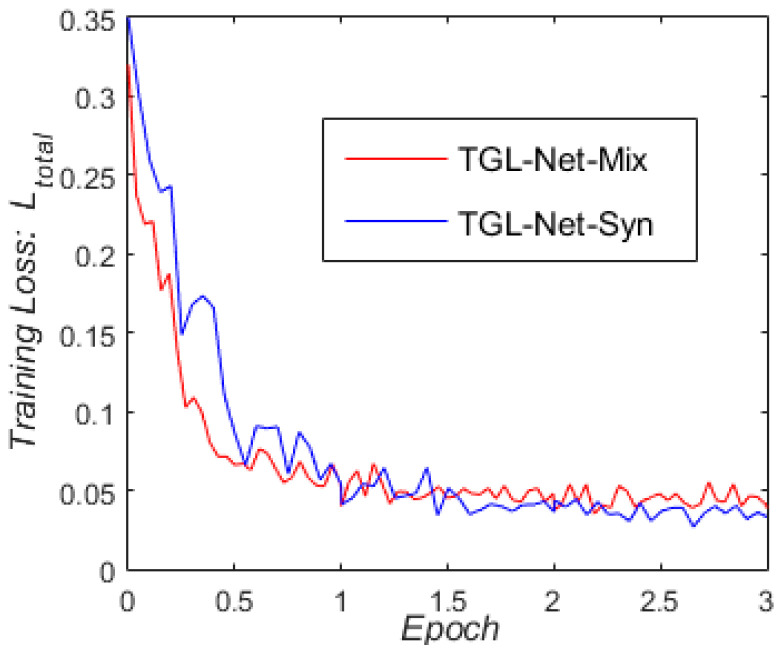
Loss curves for TGL-Net-Syn trained with only the synthetic set A, and TGL-Net-Mix trained with a combination of both the synthetic image set A and the real-world image set B.

In addition, we compared the influences of using different training sets on training efficiency. Figure 9 presents the loss curves for TGL-Net-Syn (trained by only the synthetic image set A) and TGL-Net-Mix (trained by set A and the real-world image set B) as functions of the number of training epochs. The descending loss curves indicated that real-world training sets cause TGL-Net-Mix to converge more quickly and consistently. However, both networks reached convergence after only two or three epochs.

**Figure 10:**
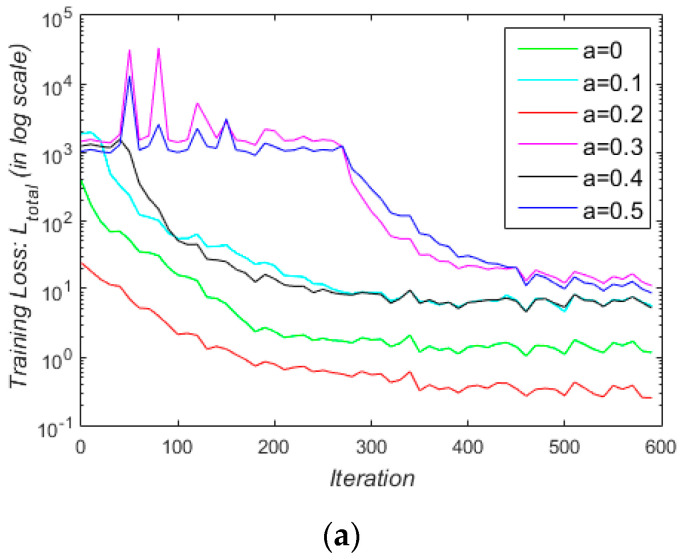

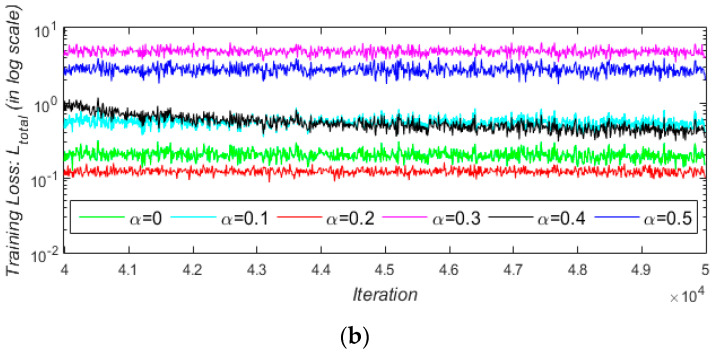
Loss curves for training TGL-Net-Mix by setting different values of loss trade-off parameter *α*, which is set from 0 to 0.5 with an increment of 0.1. For a better illustration, both vertical axes are shown in logarithmic scale. One iteration corresponds to training a batch of images with a batch size of 16. (**a**) Loss curve in iterations from 0 to 600; (**b**) loss curve in iterations from 4 × 10^4^ to 5 × 10^4^.

Furthermore, to study the effect of transmission loss on the convergence of the training process, we tested different values of *α* (0 < *α* < 0.5), which is a trade-off parameter between the transmission loss *L_T_* and the dehazing loss *L_J_* in Equation (5). Figure 10 shows the loss curves for *α* = 0, 0.1, 0.2, 0.3, 0.4, and 0.5. In the first iterations from 0 to 600 (Figure 10a), all curves descended after some changes in initial iterations, among which the training process related to *α* = 0.2 was the steadiest and the fastest. In the final iterations from 4 × 10^4^ to 5 × 10^4^ (Figure 10b), when all training processes are convergent, the red curve corresponding to *α* = 0.2 had the lowest loss values in general. Therefore, we set *α* = 0.2 to train TGL-Net-Mix.

#### 4.3.2. Downsampling and Upsampling

To demonstrate the influence of the downsampling and upsampling on the computational efficiency of the network, we trained a contrasted network TGL-ResFix, which removes the max-pooling layer and the bilinear layer in Figure 2 from the proposed TGL-Net-Mix without any other changes. Therefore, the resolution of an input image is constant across the layers in TGL-ResFix. Table 11 compares TGL-Net-Mix and TGL-ResFix for images with different resolutions in terms of the amount of computation (in GFLOPs), average run time (in seconds), and percentage of CPU consumption. With downsampling and upsampling layers, TGL-Net-Mix significantly reduced the amount of computation to approximately 15 times as much as that of TGL-ResFix. This saved approximately 80% the average run time and approximately 40% the CPU utilization compared with TGL-ResFix.

#### 4.3.3. ReLU and ELU Activations

**Figure 11:**
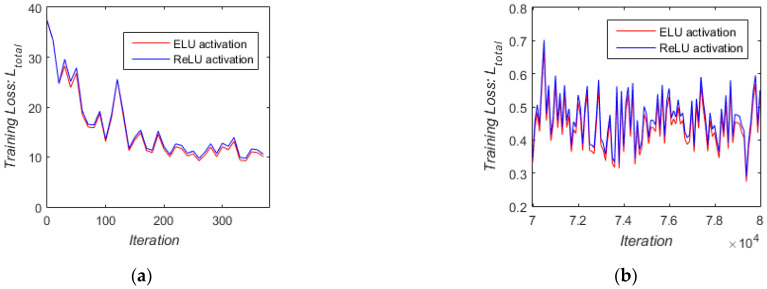
Loss curves for training the proposed network with ReLU and ELU activations, respectively. (**a**) Loss curve in iterations from 0 to 400; (**b**) loss curve in iterations from 7 × 10^4^ to 8 × 10^4^.

We further compared the effects of ReLU and ELU activation functions on network training. We trained the proposed network by replacing ELU with ReLU in Figure 2, retaining all other settings, including the same initial randomly selected network parameters. Figure 11 shows loss curves for training with these two types of activations, indicating that the value of loss training by ELU activation decreased faster in the first 400 iterations (Figure 11a) and converged to a smaller loss values in the last 10,000 iterations (Figure 11b). The results of the experiments show that ELU activation leads to faster and better convergence for training than ReLU activation.

### 4.4. Transmission Map

**Figure 12:**
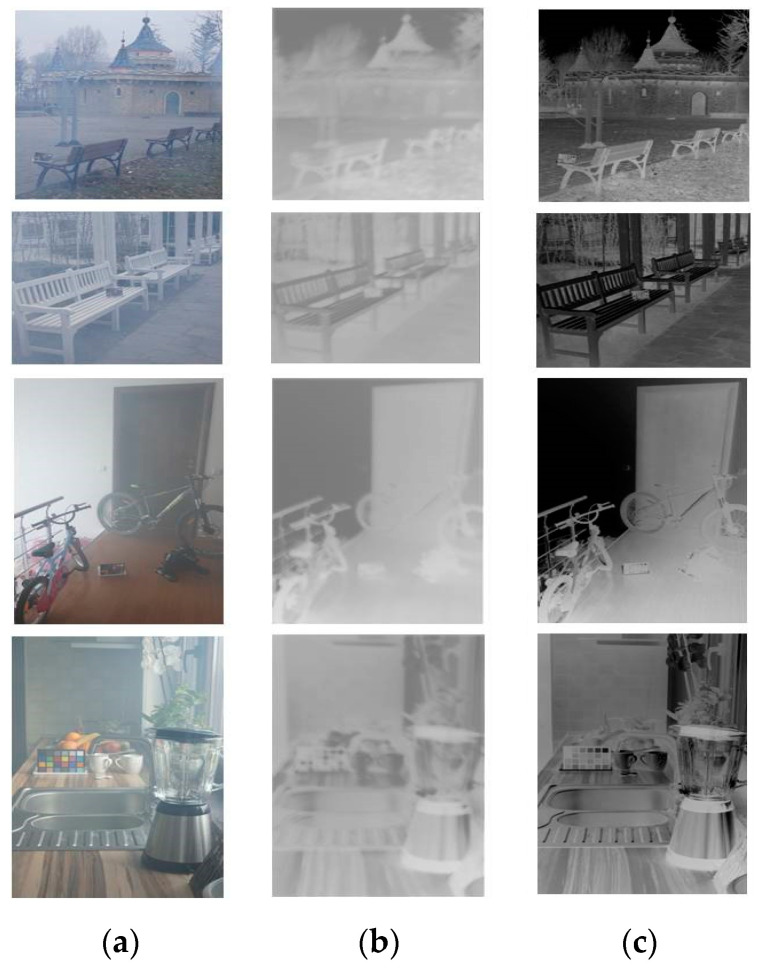
Transmission maps generated by TGL-Net (T_TGL-Net_) and F-DCP (T_F-DCP_) from hazy inputs. (**a**) Hazy inputs; (**b**) T_TGL-Net_; (**c**) T_F-DCP_.

Figure 12 presents several transmission maps generated using the TGL-Net dataset (Figure 12b) and F-DCP algorithm (Figure 12c) as references. These maps are used for computing transmission loss. The F-DCP transmission maps appear darker and clearer overall than the corresponding network-generated maps. Because TGL-Net is trained with guidance not only from transmission maps, but also from dehazed outputs, the effect of the atmospheric light A is included in the output transmission map, which makes the map blurrier and more greyish. However, our experiments reveal that using F-DCP transmission maps directly for dehazing generates vertical stripes in output images, whereas dehazed outputs from TGL-Net contain no such artifacts. In addition, DCP-based methods tend to introduce oversaturation problem and the colour distortion in white scenes, since DCP takes regions of white objects with high intensities as hazy areas (such as the sky) due to values of dark channel far from zero [12]. For instance, in Figure 12c, the sky region in the first row, white park benches in the second row, and white walls in the third and fourth rows are all displayed in black, which correspond to a value of transmission close to zero (T(x) ≈ 0 in Equation (1)). However, transmission values of these regions are much larger in Figure 12b. The reason is that TGL-Net is trained on both transmission loss and dehazing loss, therefore, to minimize the dehazing loss corrects inaccurate estimation of ground truth transmission maps to some extent. In Section 4.2, our experiments show that TGL-Net is superior to its transmission guidance, F-DCP, in terms of both quantitative and qualitative evaluations.

### 4.5. Results on Images from Other Domains

**Figure 13:**
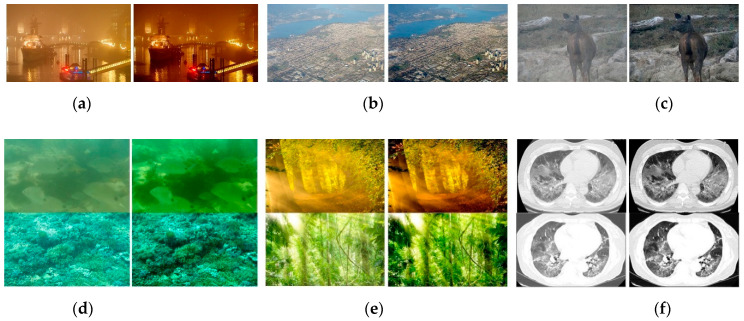
Extension to other potential applications. For each image pair, the left image is the input image and the right image is the enhanced image using the method described in this paper. (**a**) Night-time image; (**b**) remote sensing image; (**c**) low-contrast image; (**d**) underwater images; (**e**) images with halation; (**f**) CT scan images.

We performed some additional experiments to demonstrate other potential applications of image enhancement. The results are presented in Figure 13. Our network can be directly applied to night-time images, remote sensing images, and low-contrast images to enhance contrast and visibility without retraining. Additionally, if we ignore colour casting caused by light scattering, TGL-Net can effectively remove haze-like effects from underwater images (Figure 13e) and images with halation (Figure 13f), although these imaging models are significantly different. Another potential application is medical image processing. Figure 13g presents two examples of chest computed tomography scans. Their contrast is enhanced by TGL-Net, resulting in additional details for computer-assisted diagnosis.

### 4.6. Results on Challenging Cases

**Figure 14:**
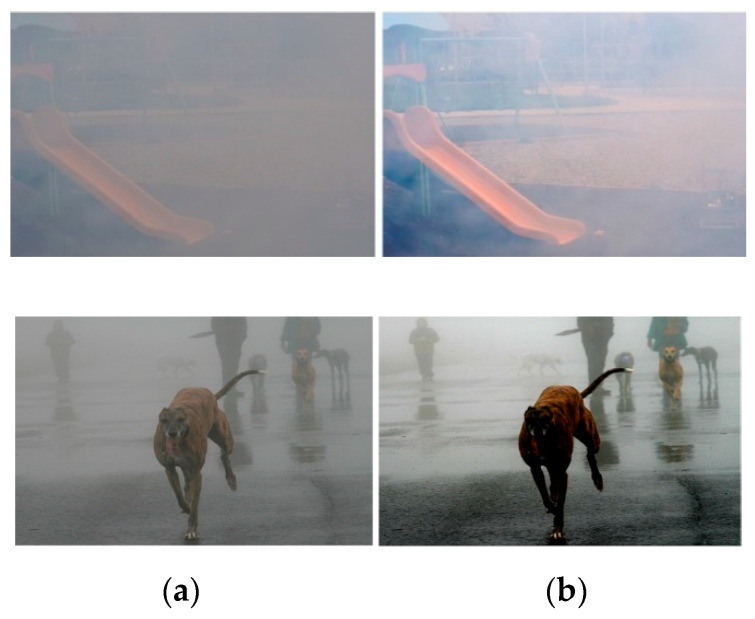
Image enhancement results on challenging cases. The left image is the input and the right image is the output. (**a**) Heavy haze; (**b**) large depth of field.

Although the proposed TGL-Net generates suitable outputs for most natural hazy images, it may fail on certain images with very heavy haze or large DOF ranges. Figure 14 presents two examples of such cases. Figure 14a presents an image with extremely dense fog from the NTIRE 2019 challenge dataset and the corresponding dehazed output generated by our network. Because TGL-Net is trained on natural images of scenes with typical degrees of haze, it is difficult for our model to produce satisfactory results for images with very heavy haze. In Figure 14b, the input image has a large DOF range from the nearby dog to the faraway objects that is completely covered by dense fog. Although our method recovers the distant scene to enhance visibility, objects close to the camera are darkened by excessive dehazing. This also occurs frequently in heavy haze scenes. Adding examples of heavy haze images to the training dataset may help to improve the performance of our network under such conditions.

## 5. Conclusions

In this paper, we proposed the TGL-Net method based on a condensed encoder–decoder structure for the fast dehazing of real images. We introduced transmission priors from a recovery-based algorithm, called F-DCP, to guide the training of our network by applying a double-error loss function combining errors from both transmission maps and dehazed images. Guidance from F-DCP enabled network training not only on synthetic data, but also on real hazy images. This method provided a feasible solution for introducing priors obtained from non-learning-based image processing techniques as guidance for training DNNs, thereby reducing the number of network parameters and improving efficiency while obtaining SOTA dehazed results. Extensive experimental results demonstrated the efficacy and efficiency of TGL-Net. The F-DCP method used in this study can be replaced with any other non-learning-based image dehazing algorithm or can be incorporated into any other transmission-guided dehazing network, which we will explore in our future work.

## Figures and Tables

**Table 1:** Summary of differences from previous dehazing networks.

Dehaze Network	Ours	DehazeNet	AOD	Cycle	PDN	GDN	PMS-Net	Joint-GAN	SSID
Published in	--	TIP	ICCV	CVPR	ECCV	ECCV	CVPR	TCSVT	TIP
Published year	2021	2016	2017	2018	2018	2019	2019	2020	2020
Reference No.	--	[6]	[8]	[9]	[10]	[11]	[12]	[13]	[14]
Symmetrical structure	✓	x	x	x	x	✓	x	✓	✓
Skip connection	✓	x	partly	partly	x	partly	partly	✓	✓
Transmission loss	✓	✓	x	x	✓	x	x	✓	x
Transmission-guided	✓	✓	x	x	✓	x	✓	✓	x
Estimate transmission from real-world images	✓	x	x	x	x	x	x	x	x
Training on real-world images	✓	x	x	✓	x	x	x	x	✓
Training on synthetic images	✓	✓	✓	✓	✓	✓	✓	✓	✓
Lightweight (compared to AOD)	✓ rank 2	✓ rank 3	✓ rank 1	x	x	x	x	x	x
Efficient (comparable to AOD)	✓ rank 1	x	✓ rank 2	x	x	x	x	✓ rank 3	x

We view a network as a lightweight and efficient model if it is comparable to all-in-one dehazing (AOD), which is verified to be an efficient model with a number of parameters of approximately 1.71 K for fast image dehazing in several papers.

**Table 2:** Architecture of the TGL-Net model.

Network Phase	Type	Input Size h × w × c	Kernels f × f × n
Downsampling	Conv	480 × 640 × 3	3 × 3 × 3
	MaxPool	480 × 640 × 3	5 × 5 × 1
Encoder– Decoder	Conv	96 × 128 × 3	3 × 3 × 5
		96 × 128 × 5	5 × 5 × 5
		96 × 128 × 5	5 × 5 × 8
	Deconv	96 × 128 × 8	5 × 5 × 5
		96 × 128 × 5	5 × 5 × 5
		96 × 128 × 5	3 × 3 × 3
	Conv	96 × 128 × 3	3 × 3 ×1
Upsampling	Bilinear	96 × 128 × 1	-
	Conv	480 × 640 × 1	5 × 5 × 1

**Table 3:** Average peak signal-to-noise ratio/structural similarity index (PSNR/SSIM) values on the NTIRE 2018 datasets.

Method/Input	NTIRE18Val-10		NTIRE18Train-60	
	PSNR	SSIM	PSNR	SSIM
Hazy Input	13.683	0.664	14.243	0.660
DehazeNet [6]	14.042	0.638	14.521	0.640
AOD [8]	15.161	0.656	15.550	0.652
F-DCP [15]	14.863	0.676	15.171	0.665
TGL-Net-Syn	15.164	0.650	15.462	0.626
TGL-Net-Mix	15.480	0.659	-	-

**Table 4:** Average values of non-reference measures in NTIRE datasets.

Method/Input	NTIRE18-20					NTIRE19-10				
	AG	IE	FADE	BIQME	PaQ2PiQ	AG	IE	FADE	BIQME	PaQ2PiQ
Hazy Input	4.17	1.77	2.81	0.45	65.30	0.93	0.92	5.91	0.35	61.86
DehazeNet [6]	5.92	2.24	1.11	0.53	64.91	1.36	1.42	3.28	0.36	62.93
AOD [8]	5.55	2.24	1.13	0.53	63.08	2.07	1.90	1.17	0.47	60.74
Cycle [9]	5.06	3.78	1.65	0.43	69.27	0.93	0.84	5.83	0.36	60.67
F-DCP [15]	8.69	3.32	1.02	0.60	67.05	3.04	1.62	4.39	0.39	65.59
TGL-Net-Mix	9.19	2.62	0.92	0.59	65.94	3.29	1.81	2.56	0.41	66.79

**Table 5:** Average values of non-reference measures on LIVE and Internet datasets.

Method/Input	LIVE-500					Internet-48				
	AG	IE	FADE	BIQME	PiQ2PaQ	AG	IE	FADE	BIQME	PiQ2PaQ
Hazy Input	11.86	6.65	1.62	0.51	68.70	9.20	5.04	2.06	0.48	68.14
DehazeNet [6]	15.20	7.91	0.80	0.56	67.96	12.32	6.13	0.99	0.56	68.29
AOD [8]	13.50	8.53	0.72	0.56	66.76	11.04	6.21	0.99	0.54	67.77
CAP [2]	15.37	6.63	0.76	0.55	67.46	13.14	5.20	0.94	0.55	67.77
PDN [10]	13.50	8.17	0.78	0.58	68.25	12.66	6.92	0.83	0.59	68.35
GDN [11]	15.82	7.81	0.58	0.54	67.19	12.38	6.72	0.75	0.56	67.59
F-DCP [15]	20.01	11.08	0.87	0.59	69.75	16.92	8.49	1.04	0.58	68.77
TGL-Net-Mix	20.22	7.88	0.59	0.54	68.13	15.93	6.29	0.91	0.54	68.40

**Table 6:** Numbers of parameters in different network models.

Models	DehazeNet [6]	AOD [8]	Bilinear-Net [38]	PMS-Net [12]	Joint-GAN [13]	RED-Net20 [21]	RED-Net30 [21]	TGL-Net (Ours)
Parameters	8.11 K	1.71 K	298.81 K	20.77 M	3.32 M	0.64 M	0.99 M	3.57 K

**Table 7:** Average run time (in seconds) on the LIVE-500 and Internet-48 testing sets.

Models	DehazeNet [6]	AOD [8]	CAP [2]	PDN [12]	GDN [13]	F-DCP [15]	TGL-Net
LIVE-500	3.362	0.609	0.928	3.541	10.726	0.815	0.088
Internet-48	3.438	0.599	1.177	4.059	13.873	0.983	0.087

**Table 8:** Average run time (in seconds) on the NTIRE 2018 dataset.

Models	DehazeNet [6]	AOD [8]	TGL-Net
NTIRE 2018	47.50	1.14	**0.046**

**Table 9:** Ablation study settings. Training set A is a synthetic haze set generated from the NYU dataset, and training set B is an augmented real haze set from the NTIRE 2018 dataset.

Models	Transmission Loss Guided?	Training Set	PSNR on NTIRE18Val-10
L-Net-Mix	x	A + B	15.40
TGL-Net-Syn	✓	Only A	15.16
TGL-Net-Mix	✓	A + B	15.48

**Table 10:** Average AG values on three testing sets.

Method	NTIRE18-20	Internet-48	NTIRE19-10
L-Net-Mix	4.77	10.93	1.73
TGL-Net-Mix	9.19	16.05	3.29

**Table 11:** Average values of non-reference measures on NTIRE datasets.

Network Model	TGL-Net-Mix		TGL-ResFix	
Resolution of Input Image	2048 × 2048	3840 × 3840	2048 × 2048	3840 × 3840
Computations (GFLOPs)	2.09	7.38	30.79	108.26
Run time (s)	0.89	2.69	4.15	15.32
CPU utilization (%)	13.5	19.9	20.5	35.7

## Data Availability

The proposed TGL-Net and some testing images can be downloaded from the link https://github.com/lizhangray/TGL-Net.
